# Fipexide (FPX), a chemical callus inducer, promotes in vitro shoot regeneration and *Agrobacterium*-mediated genetic transformation in three fruit tree species

**DOI:** 10.5511/plantbiotechnology.25.1228a

**Published:** 2026-03-25

**Authors:** Feng Dai, Masafumi Omori, Ryutaro Tao

**Affiliations:** 1 Graduate School of Agriculture, Kyoto University, Kyoto 606-8502, Japan

**Keywords:** fipexide, *FLOWERING LOCUS T*, genetic transformation, phytohormone, shoot regeneration

## Abstract

For fruit trees, inefficient transformation and prolonged juvenile phase lasting for years to decades remain two major bottlenecks for breeding and functional gene analysis. Recently, fipexide (FPX) has been identified as a novel chemical that enhances callus induction, regeneration, and *Agrobacterium*-mediated transformation. In this study, we aimed to optimize FPX treatment for improving shoot regeneration rate and transformation efficiency in European pear (*Pyrus communis*), highbush blueberry (*Vaccinium corymbosum*), and persimmon (*Diospyros kaki*). Explants were cultured on media supplemented with various concentrations of FPX for different durations. In European pear ‘La France’, treatment with 3 µM FPX for one week significantly enhanced regeneration compared with the conventional phytohormone condition, whereas higher concentrations (≥10 µM) and extended exposure inhibited regeneration. Similar patterns were observed in European pear ‘Bartlett’, highbush blueberry ‘O’Neal’ and persimmon ‘Jiro’. Furthermore, 10 µM FPX enhanced *Agrobacterium*-mediated transient GFP expression in all 3 species. We also attempted to introduce *Arabidopsis FLOWERING LOCUS T* (*FT*) into persimmon to shorten juvenile phase and induce precocious flowering. Three transgenic ‘Jiro’ lines with *AtFT* overexpression were successfully obtained using 10 µM FPX. Collectively, our findings demonstrate that FPX is a potent enhancer of regeneration and transformation in multiple fruit trees species, offering a promising strategy for accelerating breeding programs and gene function analysis in recalcitrant woody species.

## Introduction

Genetic transformation plays a crucial role in modern plant breeding, facilitating the incorporation of beneficial traits such as disease resistance, abiotic stress tolerance, and improved fruit quality. However, in many fruit trees, the inefficiency of transformation remains a major bottleneck for genetic transformation, primarily due to their strong recalcitrance to regeneration and the limited capacity for delivering foreign DNA into regenerable cells. These limitations have hindered the development of transgenic lines, thereby restricting both applied breeding efforts and gene functional studies (Birch 1997; Petri and Burgos 2005; Wada et al. 2020). Conventionally, transformation protocols rely on phytohormones such as 2,4-dichlorophenoxyacetic acid (2,4-D) and thidiazuron (TDZ) to induce callus formation and shoot regeneration. However, responses to these treatments vary considerably across genotypes and species, often resulting in poor regeneration and limiting reproducibility (Gambino and Gribaudo 2012). This has necessitated the search for alternative approaches to enhance callus induction and regeneration in fruit trees.

Besides transformation challenges, fruit tree breeding is hindered by the prolonged juvenile phase, lasting from several years to even decades. During the juvenile phase, they remain incapable of flowering or fruiting, focusing exclusively on vegetative growth. For example, 6–10 years are typically required for flowering of European pear (*Pyrus communis*), 2–3 years for highbush blueberry (*Vaccinium corymbosum*), and 5–7 years for persimmon (*Diospyros kaki*) (Hackett 1985; Meilan 1997). The extended juvenile phase poses a significant obstacle to efficient breeding, as well as to the functional analysis of genes associated with traits of reproductive organs. Hence, the development of early flowering lines, which would reduce the length of the juvenile phase, is highly desirable. One strategy to achieve the precocious flowering is overexpression of *FLOWERING LOCUS T* (*FT*), a key regulator of floral transition. Overexpression of *FT* has been successfully employed in various plant species to shorten the juvenile phase and induce the precocious flowering (Chen et al. 2025). For example, previous studies have demonstrated that precocious flowering can be induced in species such as apple (*Malus*×*domestica*), trifoliate orange (*Poncirus trifoliata*), highbush blueberry, and European plum (*Prunus domestica*) by overexpressing *FT* and its homologs (Endo et al. 2005; Song et al. 2013; Srinivasan et al. 2012; Tanaka et al. 2014). However, the efficiency of transformation in fruit trees remains genotype-dependent, and efficient transformation systems are still required to establish early flowering lines, especially in economically important cultivars.

Recent advances in chemical biology have identified fipexide (FPX) as a potent enhancer of callus induction, shoot regeneration, and *Agrobacterium*-mediated transformation (Nakano et al. 2018). FPX was originally developed as a psychoactive drug for the treatment of senile dementia (Missale et al. 1983). For application in plants, FPX was firstly identified through a chemical screening approach based on its ability to suppress hypocotyl elongation, suggesting a potential role in plant growth regulation (Nakano et al. 2018). FPX has demonstrated efficacy in promoting callus formation across diverse plant species, including Arabidopsis, rice (*Oryza sativa*), soybean (*Glycine max*), tomato (*Solanum lycopersicum*), cucumber (*Cucumis sativus*), and woody model plant poplar (*Populus* spp.) (Nakano et al. 2018). Unlike traditional phytohormone-based induction, FPX-treated calli exhibited distinct cellular characteristics and enhanced regeneration capacity (Nakano et al. 2018). Additionally, Yu et al. (2019) demonstrated that FPX has higher efficiency in shoot regeneration, using mature embryo of the model grass, *Brachypodium distachyon*, compared to conventional chemicals. Tanahara et al. (2022) achieved a 0.7% transformation frequency using 12.5 µM FPX in garden stock (*Matthiola incana*), while no transgenic plants were obtained with plant hormones.

Given its effectiveness in promoting plant regeneration and transformation, FPX represents a novel approach for fruit tree biotechnology. However, its application in fruit tree species has never been explored. To improve transformation efficiency in fruit trees, we investigated the effect of FPX on callus formation, shoot regeneration, and transformation efficiency in three fruit tree species: European pear, highbush blueberry, and persimmon. And *AtFT* overexpressing transformants were obtained in persimmon using FPX.

## Materials and methods

### Plant materials

European pear ‘La France’, highbush blueberry ‘O’Neal’, and persimmon ‘Jiro’ grown at the Kyoto Experimental Farm (Kyoto University, Kyoto, Japan) and European pear ‘Bartlett’ grown at Faculty of Agricultural and Food Sciences Farm (Kyoto Prefectural University, Kyoto, Japan) were used as plant materials.

Branches were cut into 1- to 2-cm pieces containing a single dormant bud. The explants were sterilized by immersing them in a 1% sodium hypochlorite solution containing 0.02% Tween-20 for 15 min, and then they were rinsed three times with sterile water. The sterilized buds were added and maintained in a shoot proliferation medium. For European pear and persimmon, the shoot proliferation medium consisted of Murashige & Skoog medium (MS medium, Murashige and Skoog 1962) (pH 5.8) supplemented with 5 µM zeatin, 30 g l^−1^ sucrose, and 8 g l^−1^ agar (Matsuda et al. 2005; Tao and Sugiura 1992). For highbush blueberry, the shoot proliferation medium consisted of MW basal medium supplemented with 20 g l^−1^ sucrose, 1.1 mg l^−1^ zeatin, and 6 g l^−1^ agar (pH 5.2) (Omori et al. 2025; Tetsumura et al. 2008). The MW medium contained equal volumes of MS medium and Woody Plant Medium (WPM) (McCown and Lloyd 1981).

### Optimization of FPX treatment concentration and duration

For European pear, healthy and green leaves aged 1–2 months were excised from shoots of ‘La France’ or ‘Bartlett’. For each treatment, 40–150 leaf explants were used. Three incisions were made perpendicularly to the midrib (Xue et al. 2023). The explants were placed on Nitsch & Nitsch medium (NN medium, Nitsch and Nitsch 1969) (pH 5.8) supplemented with either a combination of FPX at different concentrations, 30 g l^−1^ sucrose and 8 g l^−1^ agar, or a combination of 5 mg l^−1^ TDZ, 0.2 mg l^−1^ α-naphthaleneacetic acid (NAA), 30 g l^−1^ sucrose and 8 g l^−1^ agar as a control. The combination of phytohormones was based on published protocols that successfully produced transgenic plants in ‘La France’ and ‘Bartlett’, and is considered optimal for these two cultivars (Gao et al. 2002; Matsuda et al. 2005). The concentration of FPX was 0, 0.3, 1, 3, 10, 30, 100 or 300 µM. After cultivation for 1, 2, 4 or 6 weeks, all the explants were transferred to NN medium supplemented with 5 mg l^−1^ TDZ, 0.2 mg l^−1^ NAA, 30 g l^−1^ sucrose, and 8 g l^−1^ agar. The explants were cultured at 24°C under dark conditions for the initial 4 weeks to induce callus, and transferred to 24°C, 16-h photoperiod to induce adventitious buds from the fifth week. All media were autoclaved at 121°C for 20 min prior to use. For ‘Bartlett’, the method to induce callus and adventitious bud was the same as ‘La France’, but the concentration of FPX was 1, 3 or 10 µM, and the period of FPX treatment was 1 week.

For highbush blueberry, 50 or 150 leaf explants were used for each treatment, and about 1/3 of the tip of the leaf was cut perpendicularly to the midrib and the rest part was used as an explant. Explants were placed on MW medium (pH 5.2) supplemented with either a combination of 1, 3 or 10 µM FPX, 20 g l^−1^ sucrose, and 6 g l^−1^ agar, or a combination of 1 mg l^−1^ TDZ, 0.5 mg l^−1^ NAA, 20 g l^−1^ sucrose, and 6 g l^−1^ agar as control (Omori et al. 2021). After cultivation for 6 days, the explants were transferred to MW medium supplemented with 1 mg l^−1^ TDZ, 0.5 mg l^−1^ NAA, 20 g l^−1^ sucrose, and 6 g l^−1^ agar. The explants were cultured at 24°C under dark conditions for the initial 3 weeks to induce callus, and transferred to 24°C, 16-hour photoperiod to induce regeneration from the fourth week.

For persimmon, 36 leaf explants were used for each treatment, and a 1 cm×2 cm rectangle with the midrib in the middle was excised from leaves as an explant. Explants were placed on 1/2N MS medium (pH 5.8) supplemented with either a combination of 1, 3 or 10 µM FPX, 30 g l^−1^ sucrose, and 8 g l^−1^ agar, or a combination of phytohormone, 30 g l^−1^ sucrose, and 8 g l^−1^ agar as control (Gao et al. 2013). Two kinds of phytohormone combinations were applied: (1) 2.5 mg l^−1^ forchlorfenuron (4PU30), 0.175 mg l^−1^ IAA; (2) 2.19 mg l^−1^ zeatin, 0.175 mg l^−1^ IAA. After culture for 3 days, two groups of explants cultivated in FPX media were transferred to either 4PU30 or zeatin media. Explants were always maintained under dark conditions at 24°C.

Regeneration rate and average number of adventitious buds were documented over time. The calli that had not regenerated were excluded when taking statistics of the average number of adventitious buds. Calli that regenerated with no less than 10 adventitious buds were difficult to define and they were counted separately. Three biological replicates were used for each treatment in each experiment.

### Transient expression of GFP in leaf explants

**Figure 1:**
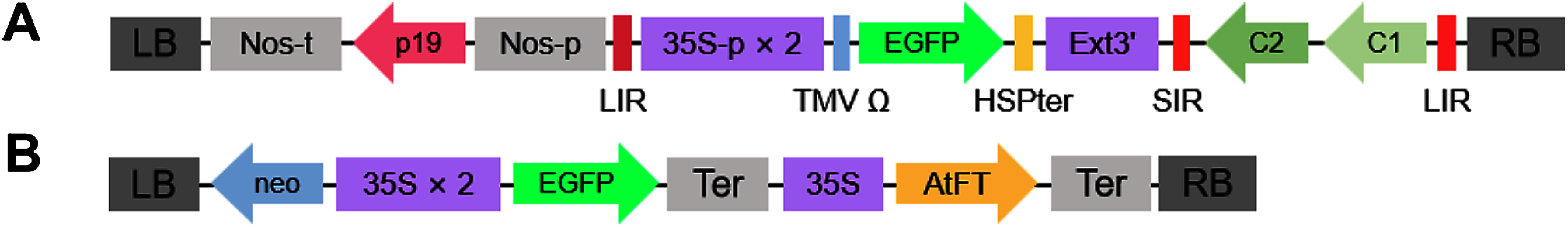
Figure 1. Schematic diagram of the T-DNA region of the vector used for transient expression of EGFP, pTKB3-EGFP (A), and vector for introducing *AtFT*, *35S::AtFT* (B). 35S-p×2: CaMV 35S promoter with double-enhanced element; TMV Ω: 5′-leader sequence of tobacco mosaic virus; EGFP: enhanced green fluorescence protein; HSPter: terminator of heat shock protein gene; Ext3′: tobacco extension gene 3′ element; LIR: long intergenic region of bean yellow dwarf virus (BeYDV) genome; SIR: short intergenic region of BeYDV genome; C1/C2: BeYDV ORFs C1 and C2 encoding for replication initiation protein (Rep) and RepA, respectively; LB and RB, the left and right borders of the T-DNA region, respectively; Nos-p and Nos-t: NOS promoter and terminator, respectively; p19: a gene-silencing suppressor gene from tomato bushy stunt virus; 35S: CaMV 35S promoter; *AtFT: Arabidopsis thaliana FT*; GFP: green fluorescence protein; *neo*: kanamycin resistance gene; Ter: terminator.

*Agrobacterium* harboring vector pTKB3-EGFP was used for GFP transient expression (Nosaki et al. 2021; Omori et al. 2023). The schematic diagram of the T-DNA region was shown in Figure 1A. This vector was introduced into *A. tumefaciens* EHA105 strain by electroporation.

For European pear and highbush blueberry, 40 and over 60 leaf explants were used for each treatment, respectively, and three wounds were cut perpendicularly to the midrib and the leaves were used as explants. For persimmon, 50 leaf explants were used for each treatment, and a 1 cm×2 cm rectangle with the midrib in the middle was excised from leaves as an explant. The explants were transformed as previously described with minor modifications for European pear (Matsuda et al. 2005), highbush blueberry (Omori et al. 2025; Song and Sink 2004), and persimmon (Tao and Sugiura 1992). After *Agrobacterium* infection, the explants were co-cultivated for 6 days on the co-cultivation medium supplemented with either phytohormone or 3 or 10 µM FPX, under dark conditions. Components of all the co-cultivation media were shown in Supplementary Method 1. GFP fluorescence was observed with a fluorescence microscope (MVX10-2/KTKS, Olympus, Tokyo, Japan) at 508 nm (blue light) after co-cultivation.

For European pear, the area of GFP fluorescence was measured using ImageJ (version 1.54g, USA). GFP expression rate and GFP fluorescence area were calculated as follows: GFP expression rate=(No. of explants expressing GFP/No. of total explants)×100 (%); GFP fluorescence area=(GFP fluorescence area/total area of the explant×100 (%). For highbush blueberry and persimmon, the number of fluorescent foci instead of area was calculated.

### Genetic transformants of persimmon with *AtFT*

*Agrobacterium* harboring vector *35S::AtFT* (Figure 1B) was used for overexpression of *AtFT* (AT1G65480) (Gao et al. 2013). The transformation methods were the same as the transient expression as described above. After co-cultivation, the explants were transferred to selection media containing 50 mg l^−1^ meropenem (Me) and 50 mg l^−1^ kanamycin (Km). The period of every step and the composition of all the media for each species were shown in Supplementary Method 2. For the initial several weeks, explants were cultured in dark conditions to induce callus. The period was 28 days for European pear, and 21 days for highbush blueberry, while explants of persimmon were always maintained under dark conditions at 24°C until regeneration occurred. After dark conditions, the explants of European pear and highbush blueberry were transferred to 16-h photoperiod at 24°C or 28°C, respectively, to induce adventitious buds.

Transgene integration and the expression of foreign *FT* were confirmed by PCR and RT-PCR for 30 cycles, respectively. Genome DNA and total RNA were extracted from leaves excised from regenerated Km-resistant shoots using cetyltrimethylammonium bromide (CTAB) method and PureLink™ Plant RNA Reagent (Thermo Fisher Scientific K.K., Tokyo, Japan), respectively. cDNA was synthesized using ReverTra Ace® qPCR RT Master Mix (TOYOBO Co., Ltd., Osaka, Japan) with gDNA Remover. Primer information for PCR and RT-PCR was shown in Supplementary Table S1. Primers for *trpR* and *DkActin* (Akagi et al. 2010; Sgamma et al. 2015), with the modified annealing temperature at 60°C to decrease nonspecific amplification. *TrpR* is a tryptophan synthesis-related gene unique to bacterium and was used to confirm the absence of *Agrobacterium* (Miano et al. 2005; Sgamma et al. 2015).

## Results

### FPX promoted regeneration of 3 species

For European pear, the callus formation and regeneration rates in ‘La France’ at FPX concentrations ranging from 0 to 300 µM across different treatment periods are observed at 12 weeks after placing the leaf explants and summarized in Table 1. Callus formation rates were 98.0% and 95.0% on media supplemented with 0.3 and 300 µM FPX, respectively, and callus formation occurred in all leaf explants under other FPX treatment conditions. In the media supplemented with phytohormone as control, 96.1% of the explants formed calli with a 2.5% regeneration rate. However, no callus formation was observed on FPX-free (0 µM) medium, indicating that FPX is effective to induce callus in ‘La France’. Regeneration was observed in media supplemented with phytohormones as well as 0.3, 1, 3, 10, and 30 µM FPX, whereas no regeneration occurred at 100 and 300 µM FPX, suggesting that relatively high concentrations of FPX inhibited regeneration in ‘La France’. Calli cultured in 3 µM FPX medium for 1 or 2 weeks exhibited significantly higher regeneration rates (8.7% and 6.0%, respectively) than that in phytohormone medium (2.5%). However, prolonged exposure to the same concentration resulted in a decreased regeneration rate. Interestingly, some calli cultured on 3 µM FPX medium for four weeks developed adventitious roots instead of adventitious buds (Supplementary Figure S1). For ‘Bartlett’, across different treatment conditions, the callus formation rate ranged from 98% to 99% (Table 2). Similar to ‘La France’, the regeneration rate in 3 µM FPX medium (28.0%) was significantly higher than that in phytohormone medium (16.0%). Regarding the average number of adventitious buds, no significant difference was observed among 1 µM, 3 µM FPX, and phytohormone media. However, the number of adventitious buds in 10 µM FPX medium was significantly lower than that in 1 µM FPX medium. The morphological characteristics of the calli and adventitious buds are shown in Supplementary Figures S2 and S3. Calli formed in phytohormone medium appeared shrunken and black, whereas those developed in FPX medium were vigorous, brown, or green. Adventitious buds induced in FPX media appeared larger and healthier than those in phytohormone medium, and leaf growth was notably faster. These findings indicate that FPX enhances the regeneration efficiency of ‘Bartlett’.

**Table 1:** Table 1. Effect of FPX on regeneration of European pear ‘La France’ after 12 weeks’ culture.

FPX concentration (µM)	FPX treatment period (week(s))	No. of explants	Callus formation rate (%)	Regeneration rate (%)
0	4	50	0	0^d^
0.3	1	100	98.0	3.0±0.5^c^
1	1	100	100	2.0±2.9^cd^
3	1	150	100	8.7±1.6^a^
	2	50	100	6.0±1.0^b^
	4	50	100	0 (roots:11.1)^d^
10	1	50	100	2.0±2.9^cd^
	4	50	100	0^d^
30	1	50	100	2.0±2.9^cd^
	2	50	100	2.0±2.9^cd^
	4	40	100	0^d^
	6	60	100	0^d^
100	4	40	100	0^d^
300	4	40	95.0	0^d^
Phytohormone	—	204	96.1	2.5±0.7^cd^

Data are means±SD of 3 biological replicates. Values with different lowercase letters are significantly different from one another at *p*<0.05 (one-way ANOVA and LSD). Culture period consisted of dark condition for 4 weeks and following photoperiod for 8 weeks.

**Table 2:** Table 2. Effect of FPX treatment on regeneration of European pear ‘Bartlett’, highbush blueberry ‘O’Neal’, and persimmon ‘Jiro’ after 12 weeks’ culture.

Cultivar	Treatment	No. of explants	Callus formation rate (%)	Regeneration rate (%)	No. of adventitious buds (buds <10)	Ratio of calli with no less than 10 buds (%)
Bartlett	Phytohormone	100	99.0^a^	16.0±3.8^b^	3.56±1.76^a^^b^	0
	1 µM FPX	50	98.0^a^	24.0±8.7^a^^b^	5.92±0.14^a^	
	3 µM FPX	100	99.0^a^	28.0±5.5^a^	4.64±0.59^a^^b^	
	10 µM FPX	100	99.0^a^	15.0±2.4^b^	3.33±1.66^b^	
O’Neal	Phytohormone	150	98.7^a^	24.7±3.1^b^	2.97±1.16^b^	0
	1 µM FPX	150	93.5^a^	24.0±2.0^b^	4.60±1.48^a^	
	3 µM FPX	150	98.0^a^	35.3±2.3^a^	4.77±0.82^a^	
	10 µM FPX	150	96.5^a^	31.3±2.3^a^^b^	4.51±1.78^a^	
Jiro	Zeatin	36	100.0^a^	11.1±4.8^e^	2.89±1.01^b^	0
	1 µM FPX (+Zeatin)	36	100.0^a^	22.2±4.8^d^	3.02±1.21^b^	0
	3 µM FPX (+Zeatin)	36	100.0^a^	11.1%±4.8 (Roots: 5.6%)^e^	3.25±1.10^b^	0
	10 µM FPX (+Zeatin)	36	97.2^a^	0^f^	—	—
	4PU30	36	100.0^a^	77.8±4.8^b^	5.25±2.50^a^	11.1^a^^b^
	1 µM FPX (+4PU30)	36	100.0^a^	83.3±0.0^a^	5.45±2.35^a^	5.6^a^^b^
	3 µM FPX (+4PU30)	36	100.0^a^	75.0±0.0^b^^c^	5.66±2.65^a^	2.8^b^
	10 µM FPX (+4PU30)	36	100.0^a^	69.4±4.8^c^	5.01±2.12^a^	13.9^a^

Data are means±SD of 3 biological replicates. Values with different lowercase letters are significantly different from one another at *p*<0.05 (one-way ANOVA and LSD). FPX treatment period: 1 week for ‘Bartlett’, 6 days for ‘O’Neal’, and 3 days for ‘Jiro’. Culture period consisted of dark condition for 4 weeks and following photoperiod for 8 weeks for ‘Bartlett’; dark condition for 3 weeks and following photoperiod for 9 weeks for ‘O’Neal’; dark condition across all time for ‘Jiro’.

For highbush blueberry, the callus formation rate and regeneration rate under 1, 3, or 10 µM FPX treatments are summarized in Table 2, and the morphological characteristics of the adventitious buds are shown in Supplementary Figure S3B. Among all treatments, the callus formation rate ranged from 93.5% to 98.7%, with no significant differences observed. The regeneration rate in 3 µM FPX medium (35.5%) was significantly higher than that in phytohormone medium (24.7%), indicating that 3 µM FPX treatment for six days is optimal for ‘O’Neal’. The average number of adventitious buds in all FPX treatments was significantly higher than that in phytohormone medium, suggesting that FPX promotes the induction of adventitious buds. Furthermore, adventitious buds formed in FPX medium were larger and exhibited faster elongation compared to those in phytohormone medium (Supplementary Figure S3B), highlighting the stimulatory effect of FPX on shoot regeneration in ‘O’Neal’.

For persimmon, explants in the 4PU30 medium showed significantly higher regeneration rate and more adventitious buds than those in the conventional Zeatin medium (Table 2). Compared to those in phytohormone medium, leaf explants under 1 µM FPX treatment exhibited significantly higher regeneration rate while those under 10 µM FPX treatment exhibited significantly lower regeneration rate. Moreover, similar to ‘La France’, some calli cultured on 3 µM FPX+Zeatin medium developed adventitious roots instead of adventitious buds (Supplementary Figure S1B).

### Effect of FPX on transient expression in 3 species

The transient expression rates of GFP fluorescence in European pear ‘La France’ and ‘Bartlett’ are presented in Supplementary Table S2. GFP expression was detected in every explant across all treatment conditions for both cultivars, with an expression rate of 100%. The GFP fluorescence exhibited in ‘La France’ is shown in Supplementary Figure S4. Although no significant difference was observed between FPX and phytohormone treatments, the largest GFP fluorescence area was recorded in 10 µM FPX medium for both cultivars.

The transient expression rates of GFP fluorescence in highbush blueberry are presented in Supplementary Table S3. No GFP expression was observed in explants co-cultivated in phytohormone medium, whereas 4.9% and 3.2% of explants exhibited GFP expression in 3 µM and 10 µM FPX medium, respectively. These results suggest that highbush blueberry is a recalcitrant cultivar for *Agrobacterium* infection with leaves as explants and that FPX treatment facilitated transformation efficiency in this cultivar. GFP fluorescence signals observed in ‘O’Neal’ were predominantly localized around the edges of the explant, rather than the three perpendicular incisions.

Transient expression rates and appearance of GFP fluorescence in persimmon were shown in Supplementary Table S3 and Supplementary Figure S4. GFP expression rate in 10 µM FPX medium was significantly higher than that in phytohormone or 3 µM FPX medium. The number of average GFP foci in 10 µM FPX was the highest but with no significant difference.

### Stable overexpression of *AtFT* in persimmon using FPX

The effect of FPX on the regeneration of persimmon ‘Jiro’ infected with *Agrobacterium* harboring the 35S::*AtFT* vector is summarized in Table 3. Callus formation was observed in 100% of explants across all media, with a significantly higher regeneration rate under 1 µM FPX treatment compared to phytohormone (Table 3).

**Table 3:** Table 3. Effect of FPX on regeneration of persimmon ‘Jiro’ infected by *Agrobacterium* harboring vector *35S::AtFT* after 12 weeks’ culture.

Cultivar	Treatment	No. of explants	Callus formation rate (%)	Regeneration rate (%)	No. of adventitious buds (buds <10) (%)	Ratio of calli with no less than 10 buds (%)	Transformation rate (%)
Jiro	Phytohormone	153	100.0	32.0±3.0^b^	5.02±2.01^a^	5.8^a^	0^b^
	1 µM FPX	54	100.0	42.6±3.2^a^	4.92±1.43^a^	5.5^a^	0^b^
	3 µM FPX	54	100.0	35.2±3.2^b^	5.43±2.51^a^	5.5^a^	0^b^
	10 µM FPX	153	100.0	29.4±5.9^b^	5.80±2.26^a^	6.5^a^	2.0^a^

Data are means±SD of 3 biological replicates. Values with different lowercase letters are significantly different from one another at *p*<0.05 (one-way ANOVA and LSD). Leaf explants were cultured in dark condition across all time.

**Figure 2:**
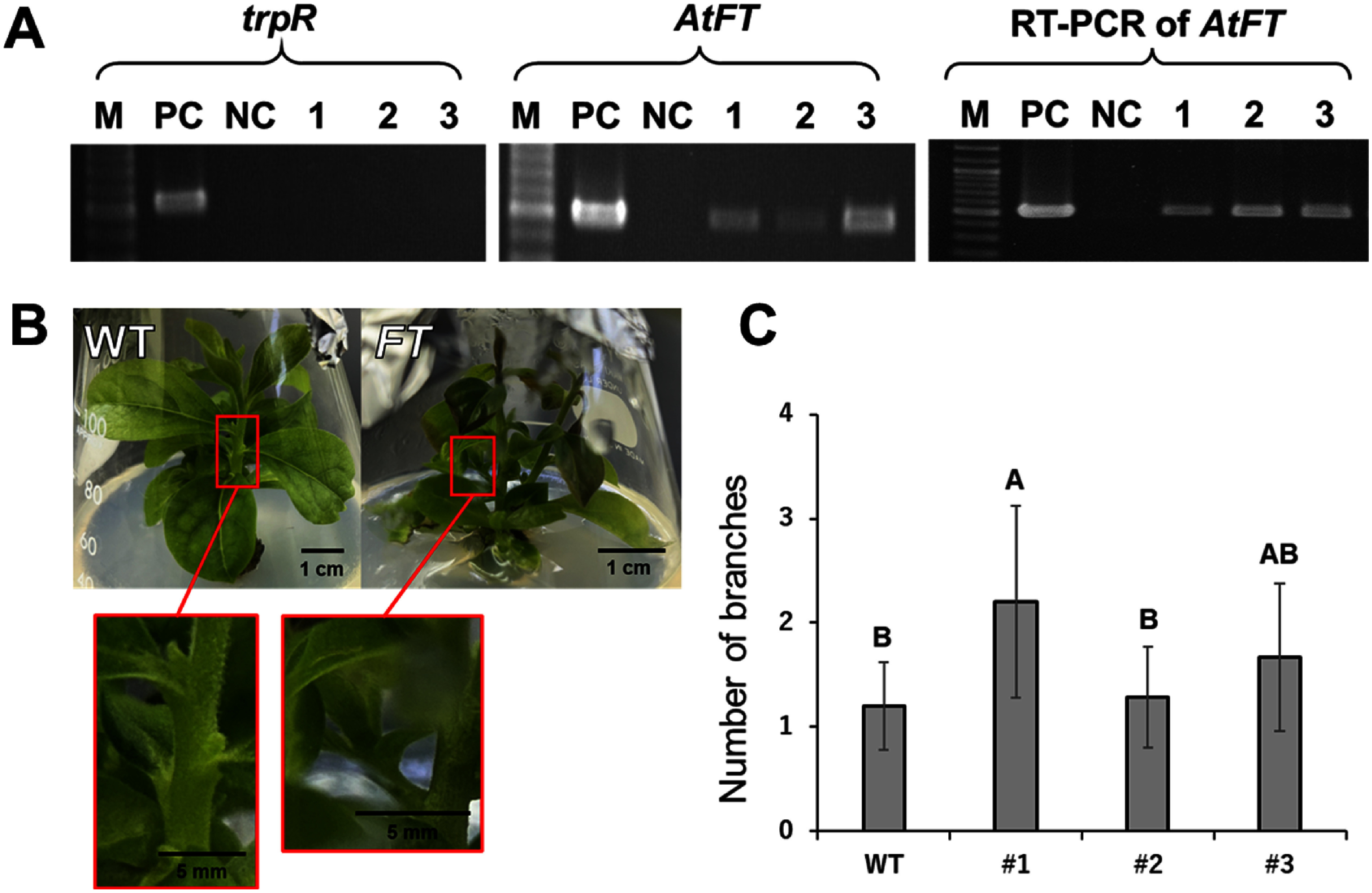
Figure 2. Phenotype of *AtFT* overexpressing transformants. Confirmation of integration and expression of *AtFT* (A). *trpR*: tryptophan repressor gene unique to *Agrobacterium*; PC: positive control, glycerol stock of *Agrobacterium* (for *trpR*), *35S::AtFT* plasmid (for *AtFT*); NC: negative control, sterilized water; WT: wild type of ‘Jiro’; number: number of lines. Phenotype of two-month-old *AtFT* overexpressing transformant (B). Axillary buds were magnified in red squares. Average number of axillary shoots of ‘Jiro’ wild type and *AtFT* overexpressing transformant #1–3 (*n*=10, 10, 7, 9) (C). Values with different uppercase letters are significantly different from one another at *p*<0.01 (one-way ANOVA and LSD).

Three independent *AtFT* overexpression lines (#1–3) of persimmon were successfully obtained using 10 µM FPX, although over 90% of the regenerated shoots were false positives. *AtFT* transgene was amplified in #1–3, while no amplification was observed for *trpR* in lines (Figure 2A). The absence of *trpR* gene indicates that the detected *AtFT* originated from T-DNA integrated into persimmon DNA rather than residual *Agrobacterium*. Furthermore, *AtFT* was also amplified using cDNA from total RNA of the transformants, confirming that the introduced gene was transcriptionally active. These results validate the stable integration and normal expression of *AtFT* in the regenerated lines, demonstrating the successful transformation and functionality of the transgene.

The phenotype of two-month-old *AtFT* overexpressing ‘Jiro’ #1 was presented in Figure 2B, while the average numbers of axillary shoots in wild type and transformant #1–3 were shown in Figure 2C. Axillary shoot numbers were calculated 2 months after subculture. Transformants of #1 exhibited a significantly higher number of lateral branches (2.20) compared to the wild type (1.20), with more rapid elongation of axillary buds.

## Discussion

Originally developed as a psychoactive agent for the treatment of senile dementia in mammals, FPX has recently emerged as a promising compound for enhancing callus formation in various model plant species. However, its efficacy in fruit trees had not been evaluated prior to this study. Here, we demonstrate that FPX significantly improved the regeneration efficiency of three fruit tree species: European pear, highbush blueberry and persimmon, highlighting its potential to stimulate organogenesis in recalcitrant species. Moreover, application of 10 µM FPX notably enhanced transient GFP expression in persimmon and enabled the successful generation of *AtFT*-overexpressing transgenic lines, indicating that FPX may facilitate *Agrobacterium*-mediated transformation by improving infection efficiency and/or T-DNA delivery.

Compared with herbal species, the optimal concentrations of FPX on regeneration of fruit trees in this research were lower, suggesting the widely varying optimal concentration across different species. On the other hand, while several species (e.g., soybean, cucumber, *Brachypodium distachyon*) tolerate or even require FPX concentrations in the 45 to 105 µM range, concentrations above the optimal level resulted in reduced callus formation efficacy in Arabidopsis root tissues and possible complete inhibition of callus formation and seedling growth in tomato (Nakano et al. 2018; Tanahara et al. 2022; Yu et al. 2019).

Interestingly, in both European pear and persimmon, calli cultured on 3 µM FPX medium occasionally developed adventitious roots instead of adventitious buds (Supplementary Figure S1). This observation aligns with the findings of Yu et al. (2019), who reported that FPX promotes adventitious root formation in *Brachypodium distachyon*. FPX-treated plants exhibited enhanced rooting and higher survival rates compared to conventional auxin-based treatments, suggesting a potential regulatory role for FPX in cell fate determination and differentiation pathways. These results imply that prolonged or high-concentration FPX treatment may shift the regenerative trajectory from shoot to root development, possibly through modulation of cell division and hormonal balance. Further investigations employing histological analyses or gene expression studies would be valuable to clarify the mechanistic basis of FPX-induced root formation and its potential utility as a rooting enhancer in woody perennials.

‘La France’ showed unique GFP expression pattern, suggesting that FPX may influence the distribution and intensity of GFP expression (Supplementary Figure S4). Considering that FPX treatment has been shown to activate cell division in Arabidopsis callus (Nakano et al. 2018), it is possible that 10 µM FPX treatment enhanced *Agrobacterium* infection in ‘La France’ by stimulating cell division and increasing the number of transformed cells (Jia et al. 2015; Lacroix and Citovsky 2013). Another possibility is that FPX may have loosened the plant cell wall or influenced cell membrane permeability, facilitating the entry of T-DNA into the nucleus. Further investigation is required to elucidate the precise mechanism by which FPX enhances transient gene expression. GFP fluorescence signals observed in highbush blueberry were predominantly localized around the edges of the explant, rather than the three perpendicular incisions (Supplementary Figure S4). It suggests the possibility that the marginal parts might have exhibited higher metabolic activity and cell division, making them more susceptible to *Agrobacterium* infection compared to the mechanically induced incisions in the central parts (Donnelly et al. 1999; Bilsborough et al. 2011).

Increased GFP fluorescence rate and the successful generation of *FT*-overexpressing transformants in persimmon suggested that 10 µM FPX treatment promoted *Agrobacterium* infection in persimmon. Relatively high FPX concentration may enhance stimulation of *Agrobacterium* uptake, but excessive FPX may lead to inhibition or other negative effects on shoot regeneration. Consequently, achieving an optimal balance between regeneration capacity and transformation efficiency essential for protocol optimization. In persimmon, GFP was observed in midribs with all different medium compositions. Higher cellular competency in the midrib may explain this localized distribution. The midrib consists of actively dividing vascular and parenchyma cells, which may have more accessible cell walls compared to the tightly packed mesophyll cells in the lamina and allow a higher competency for T-DNA uptake and transgene expression (An et al. 2014; Donnelly et al. 1999). Additionally, considering most shoots regenerated from the infected calli were false positive, further optimization of FPX concentration, co-cultivation conditions, and selection strategies are necessary to improve transformation efficiency in persimmon.

Although persimmon transformation has been reported in previous studies with efficiencies of 1.5–5.5% (Zhang et al. 2022) and 3.0% (Gao et al. 2001), reports on *FT*-overexpressing transformants remain scarce. In blueberry, transformation with *VcFT* (2.8% transformation efficiency) was significantly less successful than with a reporter gene like *GUS* (13.3% transformation efficiency) (Gao et al. 2016). This suggests that *FT* overexpression negatively impacts regeneration, making *FT* transformants difficult to obtain. In our study, we utilized FPX for the generation of *AtFT*-overexpressing transformants in persimmon and achieved a transformation rate of 2.0%.

The phenotypic characteristics observed in *AtFT*-overexpressing persimmon were consistent with plants overexpressing *FT* genes, such as tobacco (*Nicotiana tabacum*) overexpressing *GhFT* from cotton (*Gossypium hirsutum*), trifoliate orange (*Poncirus trifoliata*) overexpressing *CiFT* from *Citrus*, *Eucalyptus* overexpressing *AtFT*, and plum (*Prunus domestica*) overexpressing *populus FT*, which exhibited precocious flowering afterward (Endo et al. 2005; Klocko et al. 2016; Li et al. 2015; Srinivasan et al. 2012). This suggests that *AtFT* overexpressing persimmon may also possess the potential for early flowering, making it a promising candidate for further applications, such as using transgenic lines as rootstocks to accelerate the breeding cycle. In highbush blueberry, *Jatropha curcas*, tomato, overexpression of *FT-like* enabled the florigen from the transgenic rootstock to convey and promote flowering in non-transgenic scion (Borovsky et al. 2020; Song et al. 2019; Tang et al. 2022). Moreover, utilizing early flowering lines induced by *FT-like* or *MADS*-*box* gene made it possible to accelerate the breeding cycle of fruit trees, such as apple, European pear, and *citrus*, from decades to even less than 5 years (Endo et al. 2020; Schlathölter et al. 2018; Tomes et al. 2023). Comprehensive phenotypic and reproductive analyses on flowering time and reproductive development in *FT*-overexpressing transformants will be essential to determine their practical utility to accelerate persimmon breeding programs.

## Abbreviations

1/2N MS medium: half-strength nitrogen Murashige & Skoog medium
2,4-D: 2,4-dichlorophenoxyacetic acid
4PU30: forchlorfenuron
*At*: *Arabidopsis thaliana*
*Ci*: *Citrus*
FPX: fipexide
*FT*: *FLOWERING LOCUS T*
*Gh*: *Gossypium hirsutum*
Km: kanamycin
Me: meropenem
MS: Murashige & Skoog
NAA: α-naphthaleneacetic acid
NN: Nitsch & Nitsch
TDZ: thidiazuron
WPM: Woody Plant Medium

## Accession numbers

*AtFT*: NM_001334207.1

## Description of supplementary files

Supplementary Table S1. Primers used for confirmation of *AtFT* transformant.

Supplementary Table S2. Effect of FPX treatment on transient expression of GFP in European pear ‘La France’ and ‘Bartlett’.

Supplementary Table S3. Effect of FPX treatment on transient expression of GFP in highbush blueberry ‘O’Neal’ and persimmon ‘Jiro’.

Supplementary Figure S1. Adventitious roots formed from callus of ‘La France’ and ‘Jiro’, which had been cultivated in 3 µM FPX medium.

Supplementary Figure S2. The effect of FPX on callus formation of ‘Bartlett’.

Supplementary Figure S3. Effect of FPX on adventitious bud regeneration of European pear ‘Bartlett’ and highbush blueberry ‘O’Neal’.

Supplementary Figure S4. GFP fluorescence of transient expression shown in explants of European pear ‘La France’, highbush blueberry ‘O’Neal’, and persimmon ‘Jiro’ using FPX.

Supplementary Method 1 Composition of culture media for transient expression of GFP.

Supplementary Method 2 Culture conditions for overexpression and selection.
