# Supplementary material for: Fipexide (FPX), a chemical callus inducer, promotes in vitro shoot regeneration and Agrobacterium-mediated genetic transformation in three fruit tree species
Source: Plant Biotechnol (Tokyo). 2026 Mar 25;43(1):117–25. doi: 10.5511/plantbiotechnology.25.1228a (PMC13170794; doi:10.5511/plantbiotechnology.25.1228a)
Supplement: Supplementary Data [file plantbiotechnology-43-1-25.1228a-s001.pdf]

Supplementary files

Supplementary Table S1. Primers used for confirmation of *AtFT* transformant

Primer	Sequence (5'→3')	Annealing temperature (℃)
trpR_Forward	ACTCCTCTTACGGCCATATCG	60
trpR_Reverse	ATGCGCACCCTGATAACGAGC	
AtFT_Forward	AAGTCTTCTTCCTCCGCAG	60
AtFT_Reverse	GAGACCCTCTTATAGTAAGCAGAGTTG	
DkActin_Forward	CATGGAGAATCTGGCATCATAC	60
DkActin_Reverse	GAAGCACTGGGTGCTCTTCTG	

Supplementary Table S2. Effect of FPX treatment on transient expression of GFP in European pear 'La France' and 'Bartlett'

Cultivar	Treatment	No. of explants	GFP expression rate (%)*	GFP fluorescence area (%)**
La France	Phytohormone	40	100.0	9.8±9.3 **a**
	3 μM FPX	40	100.0	9.0±8.2 **a**
	10 μM FPX	40	100.0	13.5±13.3 **a**
Bartlett	Phytohormone	40	100.0	6.2±5.2 **a**
	3 μM FPX	40	100.0	5.5±4.9 **a**
	10 μM FPX	40	100.0	8.9±7.2 **a**

Data are means±SD of 3 biological replicates. Values with different lowercase letters are significantly different from one another at $p<0.05$ (one-way ANOVA and LSD).

*(No. of explants expressing GFP / No. of total explants)×100(%)

**(GFP fluorescence area / total area of the explant×100(%)

Supplementary Table S3. Effect of FPX treatment on transient expression of GFP in highbush blueberry 'O'Neal' and persimmon 'Jiro'

Cultivar	Treatment	No. of explants	GFP expression rate (%)	No. of GFP fluorescence points
O'Neal	Phytohormone	65	0.0±0.0 **b**	/
	3 μM FPX	61	4.9±0.1 **a**	1.33
	10 μM FPX	62	3.2±2.8 **ab**	1.00
Jiro	Phytohormone	50	26.0±1.0 **b**	8.23±4.27 **a**
	3 μM FPX	50	30.0±4.8 **b**	8.13±4.44 **a**
	10 μM FPX	50	50.0±13.9 **a**	7.40±3.57 **a**

Data are means±SD of 3 biological replicates. Values with different lowercase letters are significantly different from one another at $p<0.05$ (one-way ANOVA and LSD). Significant differences were not calculated for 'O'Neal's GFP fluorescence points because the number of samples for calculation of was too small.

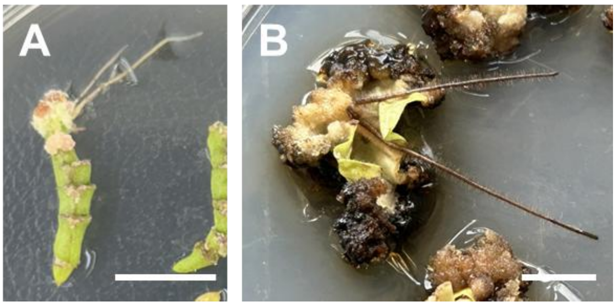

Supplementary Figure S1. Adventitious roots formed from callus of ‘La France’ (A) and ‘Jiro’ (B), which had been cultivated in 3 μM FPX medium. Scale bar = 1 cm.

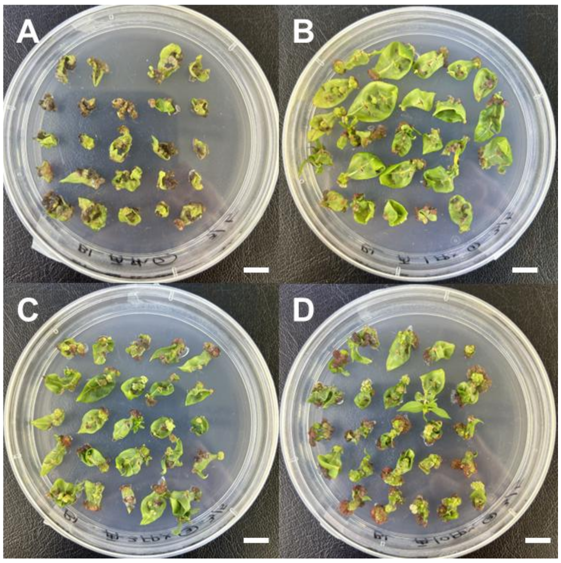

Supplementary Figure S2. The effect of FPX on callus formation of 'Bartlett'.

A: phytohormone; B: 1 μM FPX; C: 3 μM FPX; D: 10 μM FPX. Scale bar = 1 cm.

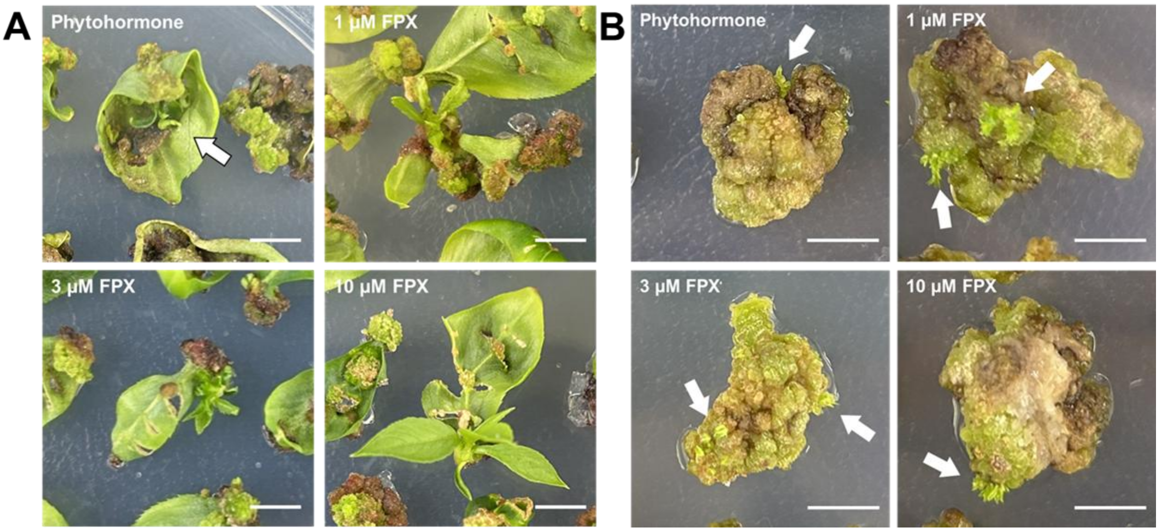

Supplementary Figure S3. Effect of FPX on adventitious bud regeneration of European pear 'Bartlett' (A) and highbush blueberry 'O'Neal' (B).

Scale bar = 0.5 cm. White arrows indicate adventitious buds.

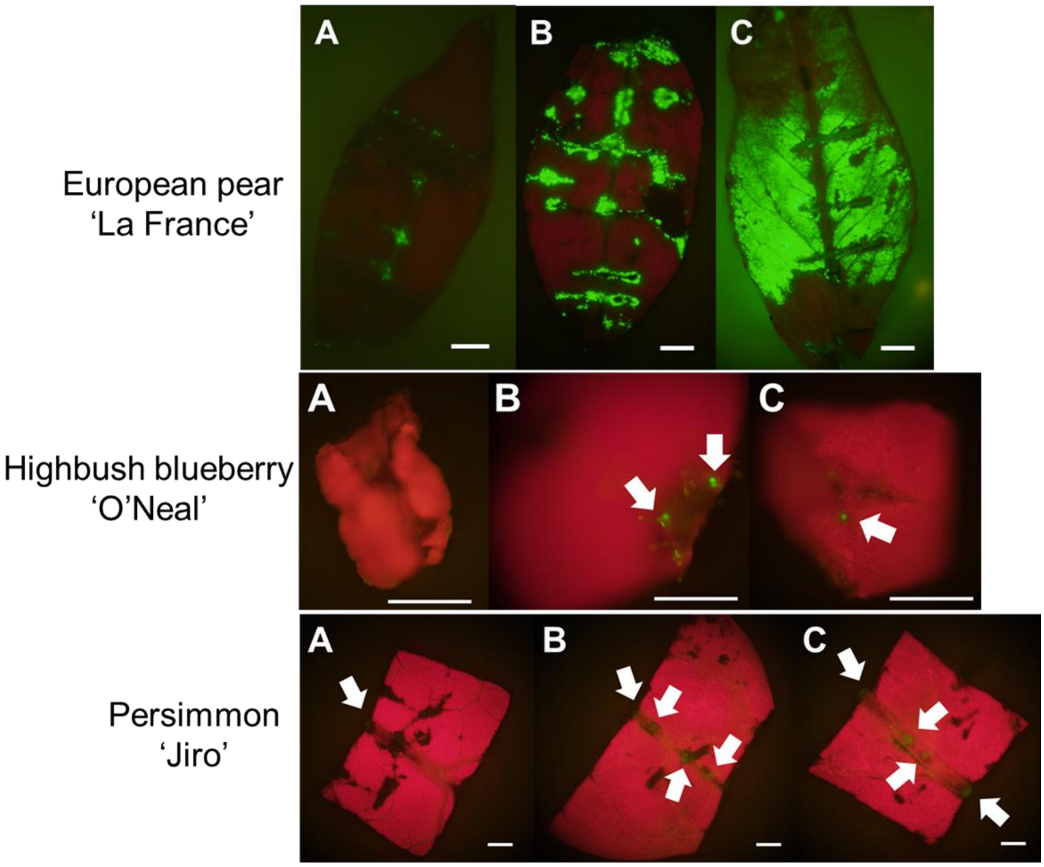

Supplementary Figure S4. GFP fluorescence of transient expression shown in explants of European pear 'La France', highbush blueberry 'O'Neal', and persimmon 'Jiro' using FPX. A: phytohormone; B: 3 μM FPX; C: 10 μM FPX. Scale bar = 1 mm. White arrows indicate GFP fluorescence.

Supplementary Method 1 Composition of culture media for transient expression of GFP

European pear:

- *Agrobacterium* suspension medium: 1/10 strength NN medium, 63 g/L sucrose, 36 g/L glucose, 0.02% (v/v) Tween-20, 100 µM acetosyringone (pH 5.2).
- Co-cultivation medium with FPX: NN medium, 3 or 10 µM FPX, 30 g/L sucrose, 4 g/L gellan gum (pH 5.2).
- Co-cultivation medium with phytohormone: NN medium, 5 mg/L TDZ, 0.2 mg/L NAA, 30 g/L sucrose, 4 g/L gellan gum (pH 5.2).

Highbush blueberry:

- *Agrobacterium* suspension medium: MW medium, 20 g/L sucrose, 0.02% (v/v) Tween-20, 100 µM acetosyringone (pH 5.2).
- Co-cultivation medium with FPX: MW medium, 3 or 10 µM FPX, 20 g/L sucrose, 6 g/L agar (pH 5.2).
- Co-cultivation medium with phytohormone: NN medium, 1 mg/L TDZ, 0.5 mg/L NAA, 20 g/L sucrose, 6 g/L agar (pH 5.2).

Persimmon:

- *Agrobacterium* suspension medium: 1/2N MS medium, 30 g/L sucrose, 0.02% (v/v) Tween-20, 100 µM acetosyringone (pH 5.8).
- Co-cultivation medium with FPX: 1/2N MS medium, 3 or 10 µM FPX, 30 g/L sucrose, 8 g/L agar (pH 5.8).
- Co-cultivation medium with phytohormone: 1/2N MS medium, 2.5 mg/L forchlorfenuron (4PU30), 0.175 mg/L IAA, 30 g/L sucrose, 8 g/L agar (pH 5.8).

All media were autoclaved at 121°C for 20 minutes.

Supplementary Method 2 Culture conditions for overexpression and selection

European pear:

- *Agrobacterium* suspension medium: 1/10 strength NN medium, 63 g/L sucrose, 36 g/L glucose, 0.02% (v/v) Tween-20, 100 µM acetosyringone (pH 5.2).
- Co-cultivation medium with FPX: NN medium, 3 µM FPX, 30 g/L sucrose, 4 g/L gellan gum (pH 5.2). Co-cultivation for 5 days.
- Co-cultivation medium with phytohormone: NN medium, 5 mg/L TDZ, 0.2 mg/L NAA, 30 g/L sucrose, 4 g/L gellan gum (pH 5.2). Co-cultivation for 5 days.
- Disinfection medium: NN medium, 5 mg/L TDZ, 0.2 mg/L NAA, 30 g/L sucrose, 8 g/L agar, 50 mg/L meropenem (Me) (pH5.8). Disinfection for 1 week.
- Selection medium: NN medium, 5 mg/L TDZ, 0.2 mg/L NAA, 30 g/L sucrose, 8 g/L agar, 50 mg/L Me, 50 mg/L kanamycin (Km) (pH5.8).

Highbush blueberry:

- *Agrobacterium* suspension medium: MW medium, 20 g/L sucrose, 0.02% (v/v) Tween-20, 100 µM acetosyringone (pH 5.2).
- Co-cultivation medium with FPX: MW medium, 3 or 10 µM FPX, 20 g/L sucrose, 6 g/L agar (pH 5.2). Co-cultivation for 6 days.
- Co-cultivation medium with phytohormone: MW medium, 1 mg/L TDZ, 0.5 mg/L NAA, 20 g/L sucrose, 6 g/L agar (pH 5.2). Co-cultivation for 6 days.
- Selection medium: MW medium, 1 mg/L TDZ, 0.5 mg/L NAA, 20 g/L sucrose, 6 g/L agar, 200 mg/L Me, 20 mg/L Km (pH 5.2).

Persimmon:

- *Agrobacterium* suspension medium: 1/2N MS medium, 30 g/L sucrose, 0.02% (v/v) Tween-20, 100 µM acetosyringone (pH 5.8).
- Co-cultivation medium with FPX: 1/2N MS medium, 3 or 10 µM FPX, 30 g/L sucrose, 8 g/L agar (pH 5.8). Co-cultivation for 3 days.
- Co-cultivation medium with phytohormone: 1/2N MS medium, 2.5 mg/L 4PU30, 0.175 mg/L IAA, 30 g/L sucrose, 8 g/L agar (pH 5.8). Co-cultivation for 3 days.
- Disinfection medium: 1/2N MS medium, 2.5 mg/L 4PU30, 0.175 mg/L IAA, 30 g/L sucrose, 8 g/L agar, 50 mg/L Me (pH 5.8). Disinfection for 1 week.
- Selection medium: 1/2N MS medium, 2.5 mg/L 4PU30, 0.175 mg/L IAA, 30 g/L sucrose, 8 g/L agar, 50 mg/L Me, 50 mg/L Km (pH 5.8).

All media were autoclaved at 121°C for 20 minutes.
